# Inversion of Forest Biomass Based on Multi-Source Remote Sensing Images

**DOI:** 10.3390/s23239313

**Published:** 2023-11-21

**Authors:** Danhua Zhang, Hui Ni

**Affiliations:** Traffic and Surveying Engineering College, Shenyang Jianzhu University, Shenyang 110168, China; zhangdh1025@163.com

**Keywords:** forest biomass, Landsat 8, Sentinel-1A, BP neural network, PSO

## Abstract

Ecological forests are an important part of terrestrial ecosystems, are an important carbon sink and play a pivotal role in the global carbon cycle. At present, the comprehensive utilization of optical and radar data has broad application prospects in forest parameter extraction and biomass estimation. In this study, tree and topographic data of 354 plots in key nature reserves of Liaoning Province were used for biomass analysis. Remote sensing parameters were extracted from Landsat 8 OLI and Sentinel-1A radar data. Based on the strong correlation factors obtained via Pearson correlation analysis, a linear model, BP neural network model and PSO neural network model were used to simulate the biomass of the study area. The advantages of the three models were compared and analyzed, and the optimal model was selected to invert the biomass of Liaoning province. The results showed that 44 factors were correlated with forest biomass (*p* < 0.05), and 21 factors were significantly correlated with forest biomass (*p* < 0.01). The comparison between the prediction results of the three models and the real results shows that the PSO-improved neural network simulation results are the best, and the coefficient of determination is 0.7657. Through analysis, it is found that there is a nonlinear relationship between actual biomass and remote sensing data. Particle swarm optimization (PSO) can effectively solve the problem of low accuracy in traditional BP neural network models while maintaining a good training speed. The improved particle swarm model has good accuracy and speed and has broad application prospects in forest biomass inversion.

## 1. Introduction

Forests are the main reservoir of terrestrial carbon and a major component of global primary productivity [1]. Forests, therefore, play a crucial role in reducing greenhouse gas concentrations and mitigating the effects of global warming [2,3]. Forest above-ground biomass (AGB) is the basis of forest carbon storage research. Accurate estimations of forest AGB can provide strong scientific support for China to achieve carbon peak and carbon neutrality [4]. In this study, tree and topographic data of 354 plots in key nature reserves of Liaoning Province were used for biomass analysis. Remote sensing parameters were extracted from Landsat 8 OLI and Sentinel-1A radar data. Based on the strong correlation factors obtained via Pearson correlation analysis, a linear model, BP neural network model and PSO neural network model were used to simulate the biomass of the study area. The advantages of the three models were compared and analyzed, and the optimal model was selected to invert the biomass of Liaoning Province. The research objectives include three aspects: (1) Which indexes of optical remote sensing and microwave remote sensing are correlated with forest biomass? (2) Which of the three models is more suitable for biomass inversion in Liaoning Province? (3) How is biomass distributed in Liaoning Province? The research results will provide a reference for exploring the application of optical and active remote sensing in forest biomass inversion.

Traditional forest biomass measurement is field measurement, which is time-consuming and laborious, and cannot be observed continuously on a large scale [5]. Remote sensing technology has many advantages in quantifying and mapping forest structures and in monitoring and mapping above-ground biomass, and it is accurate in both time and space. Therefore, a good biomass dataset containing canopy height and canopy structure can provide carbon sequestration potential for forest stocks [6]. Landsat 8 data are one of the most widely used remote sensing data types in biomass estimation. However, due to the short wavelength of the spectrum used, only forest canopy structure information can be obtained, and vertical direction parameters cannot be effectively obtained [7]. In addition, Landsat is prone to supersaturation when estimating high biomass forests [8]. Microwave remote sensing technology can obtain ground information effectively. Microwave penetration is strong, can penetrate the canopy and works all day. Synthetic aperture radar (SAR) is the most widely used radar in microwave remote sensing. SAR technology has the advantages of high spatial and temporal resolution, being all-weather, having no cloud interference and a strong penetrating ability in estimating forest above-ground biomass. This method has great potential and broad application prospects in forest biomass estimation [9]. However, different wavelengths of SAR have different saturation points. The longer the wavelength, the more sensitive it is to biomass, so long-wave radar data are more suitable for biomass estimation. Due to the high cost of large-area data acquisition with most long-wave SARs, their application in regional forest biomass estimation is limited. In 2013, the European Space Agency’s Sentinel-1A satellite began providing free high-resolution SAR data to the world. This solved the problem perfectly. Therefore, the collaborative strategy of active and passive remote sensing data can improve the accuracy of remote sensing estimations of forest above-ground biomass [10]. The application of multi-source remote sensing data fusion can better complement each other and reduce data redundancy. With the deepening of research on remote sensing technology, the collaborative application of multi-sensor remote sensing data has gradually become a trend in forest biomass estimation; in particular, the joint use of optical and radar data has become more and more popular [11]. However, there is a big problem when applying multi-source remote sensing data to forestry research, that is, data fusion; multi-source remote sensing data have different sensors or resolutions, and the data time is not correlated [12]. The diversity and extensiveness of data provide multiple possibilities for the study of forest biomass, but at the same time, it faces more challenges.

In 2005, Hyde began to use multi-source remote sensing data to invert forest parameters [13]. Kankare V et al. predicted AGB using airborne laser scanning (ALS) data and TM data collected by the Finnish National Land Survey (NLS), which began in 2008 [14]. Teja Kattenborn et al. used photogrammetric WorldView-2 data, interferometric TANTANDEM X data and hyperspectral EO-1 Hyperion data to estimate the biomass of temperate forest land near Karlsruhe, Germany [15]. Based on multi-source remote sensing data, Liu and Yaser et al. adopted the random forest regression algorithm to obtain better results [16,17]. Based on ALOS PALSAR data and other remote sensing data, respectively, Getu and Deng et al. mainly used a neural network method to estimate biomass, and achieved good inversion accuracy [18,19].

In recent years, with the rapid development of Deep Learning [20], scholars have applied it to vegetation classification. Burai et al. used SVM to identify 20 types of alkaline grassland vegetation, and the overall classification accuracy reached 82.60% [21]. Based on UAV time series images, Kwak et al. used RF and SVM to classify crops grown in Anbandegi, South Korea, and achieved good classification results [22]. Raczko et al. compared SVM, RF and ANN classifiers in vegetation classification based on aerial hyperspectral APEX images, and the results showed that ANN classifiers were superior to RF and SVM classifiers [23]. Convolutional neural networks (CNNs) have received widespread attention due to their powerful modeling capabilities and have been successfully applied in natural language processing, image recognition and other fields [24]. Kussul et al. used a convolutional neural network (CNN) to identify 11 crops in the study area, and the classification accuracy was 94.6%, which was significantly higher than the RF classification result [25]. Ji et al. used a 3D CNN framework to extract multi-temporal image information for vegetation classification; compared it with SVM, PCA and other classifiers; and found that 3D-CNN can make better use of remote sensing image phase information and improve classification accuracy [26]. In the biomedical image segmentation field, CNN-based encoding–decoding structures have exhibited superior segmentation effects in this field [27]. In the past few years, convolutional neural networks (CNNs) and graph convolutional networks (GCNs) have achieved good results in HSI classification, but CNNs struggle to achieve good accuracy in low samples, while GCNs have a huge computational cost. To resolve these issues, Bhatti et al. proposed Multi-Feature Fusion of 3D-CNN and Graph Attention Network MFFCG [28].

A neural network is a mathematical model of a distributed parallel information processing algorithm which simulates the behavior characteristics of an animal neural network. Although the combination of different bands is theoretically related to the above-ground biomass of forests, the complex environment of forests makes various statistical functions unable to express all uncertainties, so artificial neural networks have opened up a convenient, fast and reliable method for biomass estimation. This kind of network depends on the complexity of the system by adjusting the interconnection between a large number of nodes in the network to achieve the purpose of processing information. However, the BP (Back Propagation) neural network model has some problems such as slow convergence and local optimization. The performance comparison between Mahmud Iwan Solihin’s PID based on PSO (PSO-PID) and ZN-PID proves that PID tuning based on particle swarm optimization has certain advantages, so a PID controller is designed [29]. Wu Deng’s PSO algorithm can effectively improve the classification accuracy of LS-SVM and provide a new method for fault diagnosis of rotating machinery [30]. Mohamed Ahmed Mohandes used particle swarm optimization (PSO) to train an artificial neural network (PSO) to estimate the monthly average daily global solar radiation (GSR) for locations without measuring stations, using data from available stations, with excellent estimates [31]. Jin Wang used particle swarm optimization (EC-PSO) to search for energy centers and avoid energy holes in the routing protocol design of wireless sensor networks (WSNs) [32]. The above research indicates that the particle swarm optimization (PSO) method can obtain the optimal weight and deviation value, improve the prediction accuracy and training speed of the model and avoid falling into the local extreme value.

## 2. Materials and Methods

### 2.1. Overview of the Study Area

**Figure 1:**
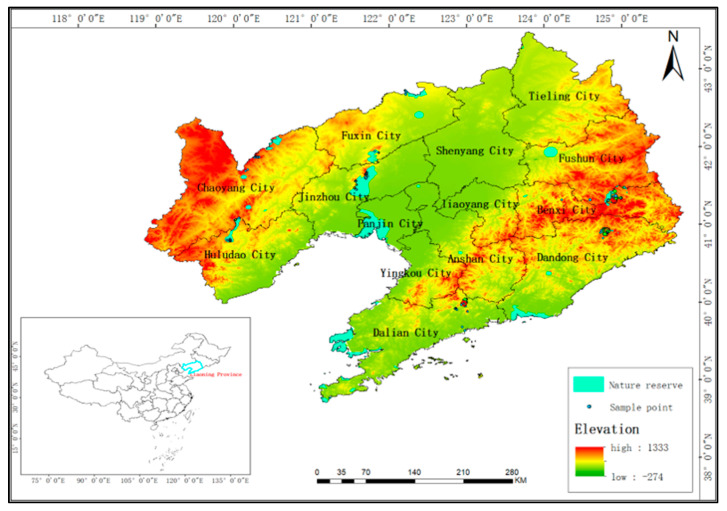
A terrain and plot distribution map of Liaoning Province.

Liaoning Province has a forest area of 6.0157 million hectares, among which arbor forests account for about 75% of the forest area, which is 4.5163 million hectares, playing an important role in the process of carbon sink. Liaoning Province is located in the southeast of China. The terrain is generally north–south, with the east and west sides tilting towards the middle; the east and west sides are mountains, tilting towards the central plain and tilting towards the tidal sea, forming a horseshoe shape. A topographic map of Liaoning Province is shown in Figure 1. It belongs to the temperate continental monsoon climate zone. The annual average temperature is 4–11 °C, affected by the monsoon climate. The annual precipitation is between 600 and 1100 mm, with uneven precipitation, wet in the east and dry in the west. The eastern part of Liaoning Province belongs to the temperate coniferous broad-leaved mixed forest belt of the Changbai flora. The main forest trees are *Picea asperata Mast*, *Abies fabri (Mast.) Craib*, *Larix gmelinii (Rupr.) Kuzen*, *Pinus sylvestris var. mongolica*, *Betula* and so on. The rest of the province belongs to the deciduous broad-leaved forest belt in the warm temperate zone, and the flora of North China is dominated by *Betula* and *Ulmus pumila.*

### 2.2. Data Source

In the study conducted by SHE X [33], vegetation indexes of five different seasons were extracted from Landsat 7 and Landsat 8 images, and the results showed that vegetation indexes in summer and autumn had a good correlation with forest biomass. Therefore, September and October, when forest vegetation grows and matures, were selected as the time period of remote sensing images.

The Landsat image was a Landsat 8 OLI remote sensing image of Liaoning Province in September and October 2019 downloaded from the Geospatial Data Cloud website (www.gscloud.cn, accessed on 1 March 2022), with a spatial resolution of 30 m × 30 m.

Sentinel-1A images were remote sensing images of Liaoning Province downloaded from the EARTHDATA website (www.earthdata.nasa.gov, accessed on 16 March 2022) in September and October 2019, with spatial resolutions of 5 m × 20 m and polarization modes of VV and VH.

The ground-measured data in this paper were the data of 354 fixed plots in the continuous forest resource inventory of Liaoning Province from 2014 to 2019. The data were from the National Ecological Science Service Center (www.nesdc.org.cn, accessed on 16 March 2022) and were divided into vegetation information and topographic information. Vegetation information included plot number, plant name, diameter at breast height, tree height, etc. Other topographic information included plot attribute table, including longitude, latitude, altitude, slope, etc.

### 2.3. Data Preprocessing

#### 2.3.1. Landsat 8 OLI Data Processing Method

Because Landsat 8 data have been geometrically and topographically corrected, pre-processing operations include radiometric calibration, atmospheric correction, image stitching and cropping. Then, the vegetation index, texture index (Mean, Variance, Homogeneity, Contrast, Dissimilarity, Entropy, Second Moment, Correlation) and band information (band2–band7) were extracted. Vegetation index is an index to measure the status of ground vegetation. In this study, seven commonly used remote sensing indices (*ARVI*, *DVI*, *EVI*, *NDPI*, *NDVI*, *RVI*, *SVAI*) were selected, and the calculation formula of each index is as follows:

(1) $$NDVI=\frac{X_{NIR}-X_{RED}}{X_{NIR}+X_{RED}}$$

Among them, *X_NIR_* refers to the near-infrared band and *X_RED_* refers to the red band.

(2) $$SAVI=\left(1+L\right)\frac{X_{NIR}-X_{RED}}{X_{NIR}+X_{RED}+L}$$

Among them, *L* is the soil adjustment coefficient; generally, its value is 0.5.

(3) $$DVI=X_{NIR}-X_{RED}$$

(4) $$RVI=\frac{X_{NIR}}{X_{RED}}$$

(5) $$ARVI=\frac{X_{NIR}-\left(2X_{RED}-X_{BLUE}\right)}{X_{NIR}+\left(2X_{RED}-X_{BLUE}\right)}$$

where *X_BLUE_* represents the blue band.

(6) $$EVI=2.5\frac{\left(X_{NIR}-X_{RED}\right)}{X_{NIR}+{6X}_{RED}-7X_{BLUE}+1}$$

(7) $$NDPI=\frac{X_{NIR}-\left(0.74X_{RED}+0.26X_{SWIR}\right)}{X_{NIR}+\left(0.74X_{RED}+0.26X_{SWIR}\right)}$$

where *X_SWIR_* stands for short-wave infrared.

#### 2.3.2. Sentinel-1A Data Preprocessing Method

The processing of Sentinel-1A images, based on the official open-source software SNAP7.0 developed by the European Space Agency, involves a series of preprocessing steps aimed at enhancing data quality. These steps encompass track correction, thermal noise removal, radiation calibration, speckle filtering, topographic radiation correction and geocoding, as well as geometric correction. Subsequently, SAR backscatter coefficient extraction and texture information analysis (Mean, Variance, Homogeneity, Contrast, Dissimilarity, Entropy, Second Moment, Correlation, ASM, MAX, Energy) are performed to derive remote sensing feature factors associated with biomass.

#### 2.3.3. Calculation of Tree Biomass in Sample Plot

The above-ground biomass per plant was calculated using the allometric growth equation proposed by Dong Lihu [34].

(8) $$W=a\cdot D^{b}\cdot H^{C}$$

In the formula, *W* is the above-ground biomass, *D* is the diameter at breast height and *H* is the height of the tree. *a*, *b* and *c* are the parameters of the model, and their values are set to 0.0470, 2.1181 and 0.7088, respectively.

#### 2.3.4. Correlation Analysis of Remote Sensing Factors and Biomass

It is very important to select highly correlated independent variables to estimate biomass. Adding unnecessary variables to the model will increase the calculation and affect the stability of the model, but if the variables are too few, it will not be conducive to the correct construction of the model [35]. Therefore, we conducted Pearson correlation analysis to screen out the independent variables significantly correlated with biomass. The correlation coefficient is calculated as follows:

(9) $$r=\frac{\sum_{i=1}^{n}\left(x_{i}-\bar{x}\right)\left(y_{i}-\bar{y}\right)}{\sqrt{\sum_{i=1}^{n}{\left(x_{i}-\bar{x}\right)}^{2}}{\left(y_{i}-\bar{y}\right)}^{2}}$$

In the above formula, *x_i_* representsthe *i*-th *x* variable, *y_i_* represents the *i*-th *y* variable and *x* and *y* represent the average value of the variables. The absolute value of *r* indicates the degree of correlation. When *r* is positive, it means positive correlation; when *r* is negative, it means negative correlation.

By removing outliers, 162 plots of biomass and their corresponding correlation factors were screened out from the samples. Pearson correlation statistical analysis was performed on the sample biomass in SPSS 26 software, and finally the correlation between tree biomass and various factors was obtained.

#### 2.3.5. Build a Linear Regression Model

Variables significantly correlated at the level of 0.01 (two-sided) were selected, and the traditional stepwise regression method was used in SPSS 26 software to construct the biomass recovery model. F test and *t* test were used to verify the accuracy of the model. R^2^ represents the square of the correlation coefficient of the regression equation, reflecting the goodness of fit between sample data and regression equation. The closer R^2^ is to 1, the better the regression equation model is. Meanwhile, the model was tested by using the measured data as the true value of the sample.

#### 2.3.6. Establish BP Neuron Network Model

The simple idea of BP neural network is as follows: the first layer is the input layer of the neural network, which acts on the neurons of the second layer; the second layer is the hidden layer, where neurons transmit stimuli to each other. The hidden layer can have multiple layers; the third layer is the output layer, and the neuron stimulation is transmitted to the outside world after multiple layers. The artificial neural network model has the advantage of high precision, but its disadvantage is that the simulation process is similar to the “black box” operation, and the internal mechanism of the model is difficult to explain well. When the artificial neural network is applied to estimate forest biomass, the input variables are remote sensing data, slope, altitude, canopy density and other factors, and the output variables are the forest biomass at the corresponding position of the pixel for modeling. Due to the strong nonlinear fitting ability of single hidden layer BP neural networks, they were used for biomass estimation. The number of neurons in the input and output layers was determined, and the number of hidden layers was required. For a single hidden layer neural network, the more neurons in the hidden layer, the stronger the nonlinear ability of the model, but the generalization error of the model will increase. The determination of the number of neurons in the hidden layer was crucial. Usually, increasing the number of hidden layer nodes can improve the output accuracy of the BP network, while also making it more complex and difficult to control the training time. Currently, the number of hidden layers is usually set according to the following formula:

- $\sum_{i=0}^{n}C_{M}^{i}>k$, where *k* is the set size of input data, *M* and *n* are the number of hidden layer and input layer nodes, respectively. If *i* > *M*, specify $C_{M}^{i}$ = 0;
- $M=\sqrt{n+m}+a$, where *m* is the number of network output layer nodes, *n* is the number of network input layer nodes and *a* is a constant between [0 and 10];
- $M=\log_{2}n$, where *n* is the number of input layer nodes.

On the basis of meeting the accuracy requirements, this study explored how to reduce the number of hidden layer neurons as much as possible to reduce the computational complexity of the model. In practical applications, considering the complexity and nonlinear factors of the problem studied, the number of hidden layer nodes is often determined with “trial and error”, that is, the number of hidden layer nodes corresponding to the minimum output error of the neural network is selected. The specific method was tested one by one in Matlab from the initial 5 hidden layer nodes to 20 hidden layer nodes. The datasets were randomly divided into training sets, validation sets and test sets according to a ratio of 70%, 15% and 15%. For the training set, by comparing the three algorithms in the neural network library of Matlab 2020a software, the neural network model based on the trainlm algorithm is finally used for calculation, and the measured data are used as the true value of the sample to test the model.

#### 2.3.7. Establishment of BP Neural Network Model Improved by Particle Swarm Optimization

Particle swarm optimization (PSO) has the advantages of fast convergence speed, few parameters, a simple algorithm and easy implementation. Particle swarm optimization (PSO) simulates the predation behavior of birds. Imagine a scenario where a flock of birds randomly searches for food. There is only one piece of food in this area. All the birds do not know where the food is. But they know how far the food is from their current location. So, what is the best strategy for finding food? The easiest and most efficient way is to search the area around the bird that is currently closest to the food. Throughout the search, the flock lets other birds know where it is by passing messages to each other. Through such cooperation, they can judge whether the optimal solution has been found, and at the same time convey the information of the optimal solution. For the whole flock of birds, eventually the whole flock of birds can gather around the food source, that is, find the optimal solution.

In PSO, the solution to each optimization problem is a bird in the search space. We call them “particles”. All particles have a fitness value determined by the optimized function, and each particle has a velocity that determines the direction and distance they fly. Then, the particles follow the current optimal particle to search in the solution space. PSO is initialized as a group of random particles (random solutions). The optimal solution is then found through iteration. In each iteration, the particle updates itself by tracking two “extrema”. The first is the optimal solution found by the particle itself, which is called the individual extremum pBest. Another extremum is the optimal solution currently found by the entire population, and this extremum is the global extremum gBest. In addition, instead of the whole population, only a part of it can be used as the neighbor of the particle; then, the extremum among all the neighbors is the local extremum.

There are two properties of particles: velocity and position. Velocity represents the direction and distance the particle will move in the next iteration, and position is a solution to the problem being solved.

The algorithm has 6 important parameters: the position of the i-th particle; the velocity of the i-th particle (the distance and direction the particle travels); the optimal position of the i-th particle (individual optimal solution); the optimal position of the group search (the group optimal solution); the fitness value of the optimal position searched by the i-th particle (the value of the optimization objective function); the adaptive value of the optimal location searched by the group.

The specific algorithm flow is as follows:

(1) Initialize a group of particles (the group size is N), including random positions and velocities;
(2) Evaluate the fitness of each particle;
(3) For each particle, compare its fitness value with the best position pbest it has passed, and if it is better, use it as the current best position pbest;
(4) For each particle, compare its fitness value with its best position gbest, and if it is better, use it as the current best position gbest;
(5) Adjust particle velocity and position;
(6) If the end condition is not met, go to step 2.

The iteration termination condition is generally selected as the maximum number of iterations and/or the optimal position searched so far by the particle swarm meets the predetermined minimum adaptation threshold according to the specific problem.

#### 2.3.8. Spatial Biomass Mapping

Through the comparison of the above modeling methods, the optimal model based on active and passive remote sensing data was selected to invert the above-ground forest biomass in Liaoning Province, and a map was made.

## 3. Results

### 3.1. Plot Data Processing

**Figure 2:**
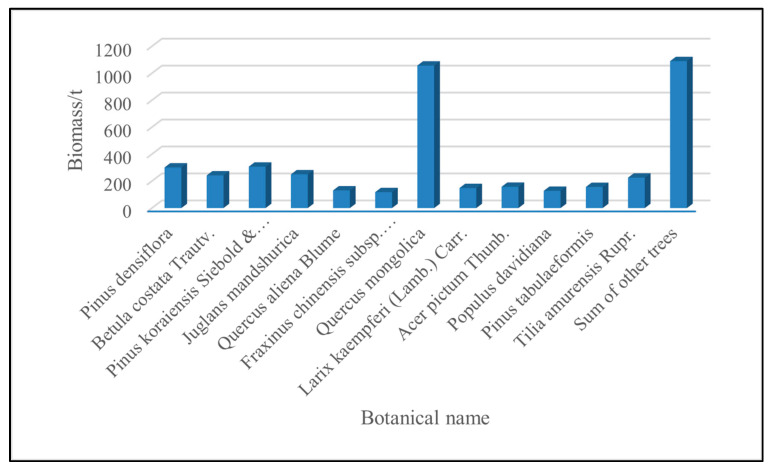
Biomass distribution map of various tree species.

After calculating the above-ground biomass of a single plant in various places, the biomass of the same tree species was added together to obtain a histogram of the biomass distribution of each tree species, and the results are shown in Figure 2. Among them, *Quercus mongolica* accounted for about 24% of the biomass in the study area, and *Populus davidiana*, *Quercus aliena Blume* and other major tree species accounted for about 3–7%, respectively. The other tree species accounted for about 25% of the total biomass in the study area.

### 3.2. Correlation Analysis of Remote Sensing Factors and Biomass

Because the optical remote sensing band information records the information received by the sensor through electromagnetic radiation, the vegetation index is a measure of surface vegetation status. Texture feature information is the structural feature formed by the regular distribution or change in color in an optical image. In optical images, texture reflects the change law of an image’s gray value or color. The SAR backscattering coefficient contains abundant information on ground objects. Therefore, these remote sensing parameters were extracted and three variables, namely forest elevation, slope and canopy, were added. There were 86 variables in total. Their correlation statistics with forests are shown in Table 1.

The results showed that 35 feature factors extracted from Landsat 8 OLI images were correlated with the forest biomass of the plot (*p* < 0.05), and 22 texture features were correlated with the forest biomass of the plot (*p* < 0.05). The Landsat 8 band2 had the highest correlation, with a correlation coefficient of −0.342. Among the vegetation index, NDPI had the highest correlation with biomass, and the correlation coefficient was 0.323. Among the characteristic factors extracted from Sentinel-1A radar images, seven factors were correlated with biomass (*p* < 0.05), and the other five factors were negatively correlated, except entropy. In other aspects, the correlation coefficients of altitude and canopy density with biomass were 0.404 and 0.328, respectively.

In summary, a total of 44 factors were correlated with biomass, among which 21 factors were strongly correlated with biomass at the (*p* < 0.01) level. Because the following three models need appropriate variables, too many variables will increase the amount of calculation and affect the stability of the model, but too few variables will not be conducive to the correct construction of the model. At the same time, a too strong or too weak correlation between variables will also affect the construction of the model. Therefore, factor 21, which showed a strong correlation at the level of 0.01, was screened and put into three models.

### 3.3. Linear Regression Model Processing Results

The results of the regression estimation model are shown in the table below (Table 2):

The *t*-test significance levels of B6Mean, VVEntropy and constant in the model were greater than 0.05; the significance levels of other variables’ *t*-tests were less than 0.05; and the F-test significance level of the model was less than 0.001 for the whole model, indicating that the regression model passed the test. By comparing the absolute value of the standard coefficient, it can be found that RVI has the greatest impact on biomass, but the absolute value of the Pearson correlation test coefficient of RVI on biomass is not the largest, and the Pearson correlation test does not match the standard coefficient. On the one hand, this shows that the data distribution does not satisfy the normality assumption, and on the other hand, it shows that there is a linear correlation between the regression coefficients of the model. Therefore, regression model results use unstandardized coefficients.

(10) $$\begin{array}{c} \;Biomass=-243.422+0.09*Altitude+125.943*Canopy\;closure-156.917*ARVI+73.454*EVI\\ +148.34*RVI+34.447*VVEntropy+24.306*B6Mean-245.049*B6 \end{array}$$

**Figure 3:**
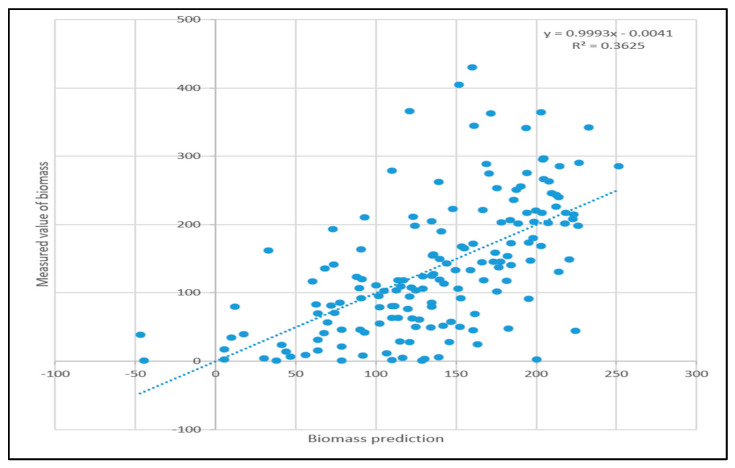
A graph comparing the results of the linear model test set with the true values.

Through linear model prediction, the predicted value of forest biomass was compared with the real value, as shown in Figure 3.

The linear model was used to measure the biomass of the sample plot, and the measured data were used as the real value of the sample plot to test the model. The comparison between the predicted value and the actual value of forest biomass in the test set is shown in Figure 3, and the coefficient of determination is 0.3625. The maximum relative error of linear simulation is 209.61 and the minimum relative error is −37.35. After disposing the values with relative errors greater than 100% as outliers, the average deviation of the predicted values is calculated to be 32%.

### 3.4. BP Neuron Network Model Processing Results

**Figure 4:**
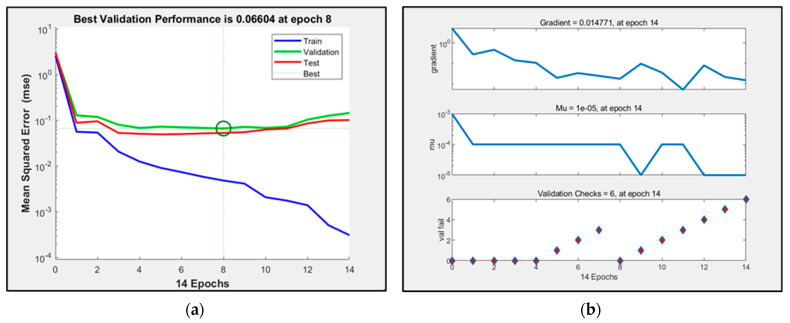
(**a**) Schematic diagram of the trainlm algorithm corresponding to network performance; (**b**) schematic diagram of the trainlm algorithm corresponding to the network training state.

**Figure 5:**
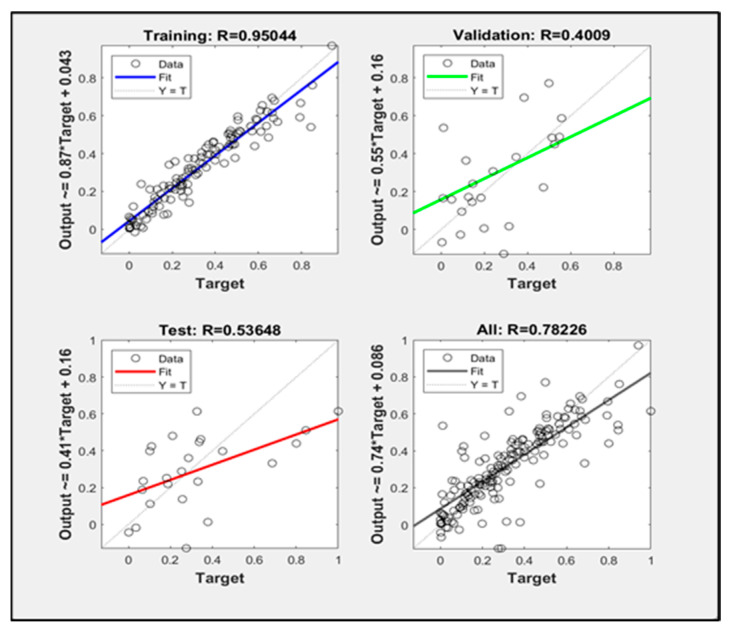
Schematic diagram of trainlm algorithm corresponding to network regression analysis.

The trainlm algorithm uses the Levenberg–Marquardt algorithm, and the training speed is faster when the amount of training data is small. The performance plots, training parameter values and regression analysis of the simulated neural network are shown in Figure 4 and Figure 5.

After many experiments, it was found that when the number of hidden layers is set to 10 and the training iteration is set to the eighth generation, the mean square error of the verification set is the smallest, which is 0.06604, and the training result is the best. The coefficient of determination is 0.78226.

**Figure 6:**
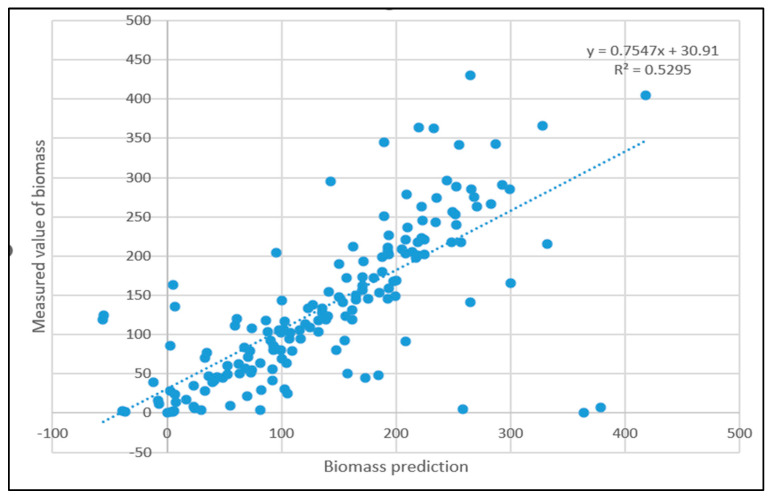
A comparison of the results of the neuronal network model test set with the true values.

The BP neural network model was used to invert the biomass, and the prediction value of the forest biomass in the test set was compared with the real value, as shown in Figure 6.

As Figure 6 shows, the coefficient of determination between the predicted value and the real value was 0.5295, the maximum relative error between the network prediction value and the measured biomass value was 2539.94, the minimum relative error was −1.63 and the rest of the test data had small errors. After treating the value with a relative error greater than 100% as an outlier and discarding it, the average deviation of the predicted value was calculated to be 30%, which can well invert the value of biomass, indicating that there was a nonlinear relationship between tree biomass and related factors.

### 3.5. Particle Swarm Optimization Algorithm Improves BP Neural Network Model Processing Results

**Figure 7:**
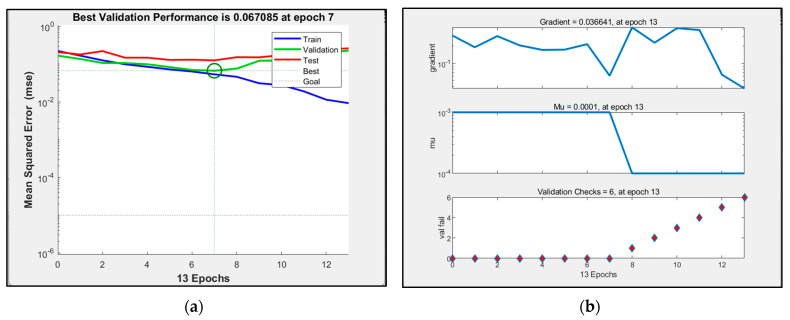
(**a**) Schematic diagram of network performance corresponding to PSO algorithm. (**b**) Schematic diagram of PSO algorithm corresponding to network training state.

**Figure 8:**
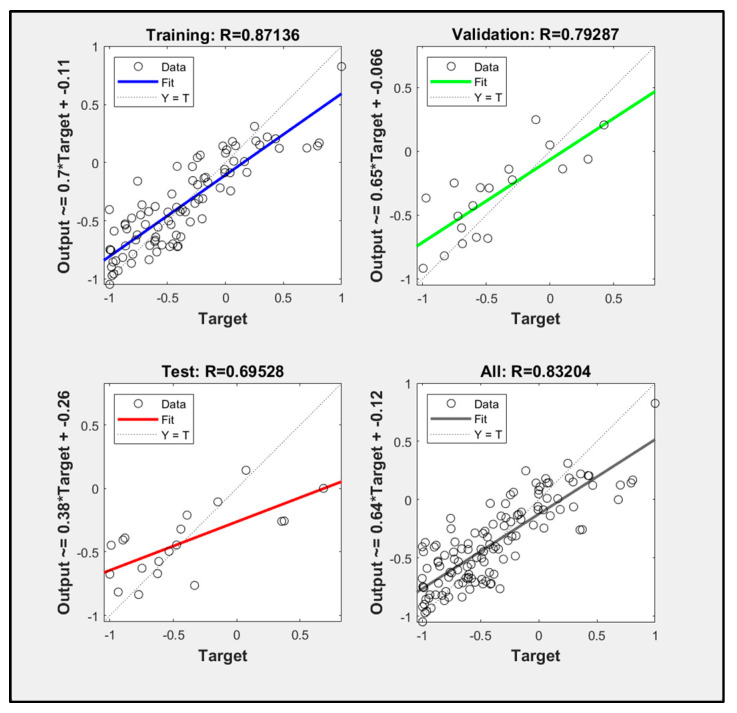
Schematic diagram of network regression analysis corresponding to PSO algorithm.

When the number of hidden layers was set to 10, the data fitting value of the PSO algorithm improved neural network model training reached 0.8320, and the simulation effect was better than that of the BP neural network. The training results are shown in Figure 7 and Figure 8.

**Figure 9:**
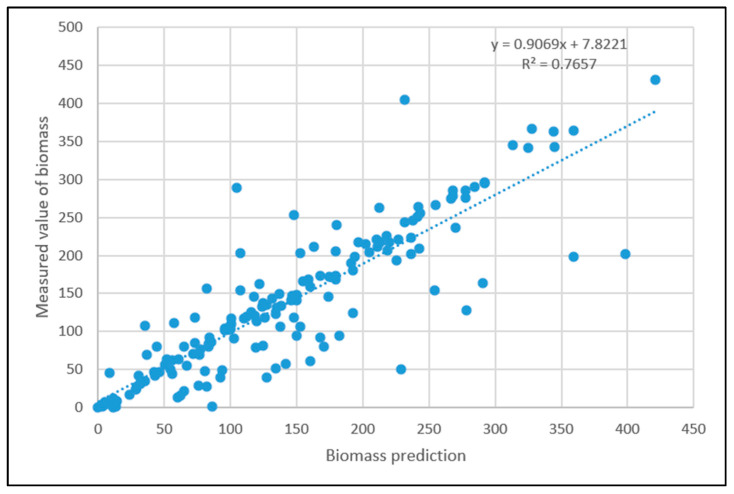
A comparison of the results of the PSO-improved neural network model test set with the real values.

The BP neural network model improved by PSO was used to invert the biomass, and the predicted value of forest biomass in the test set was compared with the real value, as shown in Figure 9.

The neural network model improved by the PSO algorithm was used for biomass inversion, and the coefficient of determination between the model’s predicted value and the real value was 0.7657, which was higher than that of the BP neural network model. The maximum relative error between the network prediction value and the measured biomass value was 74.61, the minimum relative error was 0, and the rest of the test data had small errors. After treating the values with a relative error greater than 100% as outliers and discarding them, the average deviation of the predicted value was calculated to be 19%, which can well invert the biomass value, indicating that the improved BP neural network model was better than the BP neural network model.

### 3.6. Results of Spatial Biomass Mapping

**Figure 10:**
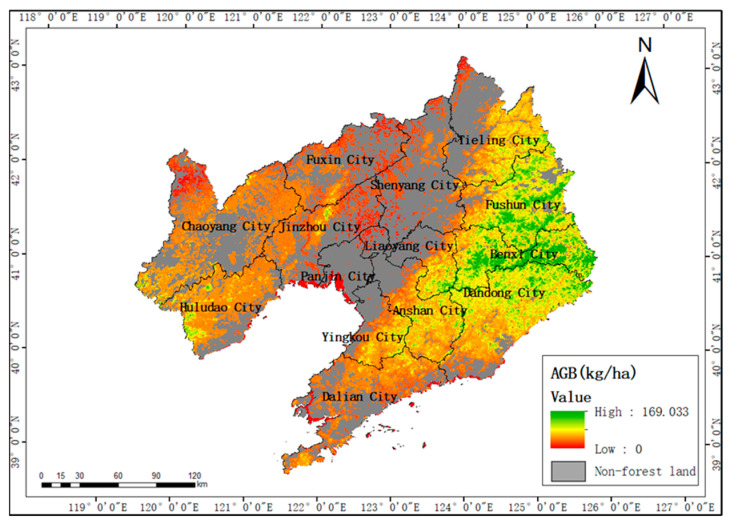
Spatial distribution of above-ground biomass.

Through the comparison of the above modeling methods, the PSO model based on active and passive remote sensing data with the best performance was finally selected to invert the above-ground forest biomass in Liaoning Province, and a biomass spatial distribution map as shown in Figure 10 was obtained.

As can be seen from Figure 10, the areas with high above-ground forest biomass in Liaoning Province are mainly distributed in the southeast and southwest areas with high altitudes and steep slopes, while the areas with low biomass are mainly concentrated in the plain areas with low altitudes and gentle slopes. The spatial distribution trend of above-ground forest biomass is consistent with the topographical features and social and economic conditions of the study area.

## 4. Discussion

The traditional stepwise regression method was used for modeling, the R^2^ between the predicted value and the actual value after adjustment of the linear model was only 0.3625, and the Pearson correlation coefficient between each band of Landsat 8 and Sentinel 1A VV and VH polarization mode was less than 0.36. The accuracy of the model can be significantly improved by adding vegetation index data and texture index data, which is consistent with many research results [36,37]. At the same time, when the elevation data were input into the model, the dynamic elevation gradient of the study area had a greater impact on biomass, which is consistent with the research results of Olimann and Gregory [38]. Each band of Landsat 8 (band2–band7) was negatively correlated with biomass, and the other vegetation indices except the enhanced vegetation index were positively correlated with biomass, which was consistent with the research results of Guo Zhihua [39]. The correlation coefficient between the band2 value and biomass was the highest, which may be because the chlorophyll in green plants absorbed the most light, making the spectral curve of reflected plants at the lowest point in the spectral range. NDPI had the highest correlation coefficient among all vegetation indices. NDPI is the ratio of the difference between the sum of near-infrared light and red light and short-wave infrared light to the difference between the sum of near-infrared light, red light and short-wave infrared light. The main absorption band of chlorophyll is red light. The lower the red light brightness value, the lower the red light reflectivity, the higher the chlorophyll content and the larger the biomass.

The running time and model determination coefficient R^2^ of the three models were compared, as shown in Table 3. It can be seen that the model training speed based on the linear stepwise regression method is slightly faster than that of the BP neural network model, but the model accuracy is lower. The BP neural network model has high prediction accuracy and short prediction time, indicating that the nonlinear model is more suitable for the inversion of tree biomass in Liaoning Province. However, the prediction value of the improved PSO neural network model is better than that of the BP neural network model. Although the time is longer, it is also within the acceptable range.

On the other hand, some output results were quite different from the measured value of biomass. In the validation of linear model results, the measured biomass distribution range was 0.56 kg/hm^2^~430 kg/hm^2^, and the scattered points were evenly distributed on both sides of the line in a ratio of approximately 1:1. However, the distance between the scattered points and the line was relatively large, indicating a large deviation in biomass prediction and a large coefficient of determination. The decision coefficient value of 0.5295 also reflected this point. There were outliers such as (0.609, 163.863) in the model, which cannot be explained by the BP neural network model. The reasons for this phenomenon may be as follows:

(1) This experiment was only a preliminary study on a forest estimation model, and there were still some shortcomings and deficiencies in the specific technical processing. More parameter characteristics would be needed for further analysis in order to better understand the impact of biological factors on factors of quantitative inversion accuracy;
(2) Due to limited conditions, the number of training data samples used in this experiment was small, which made the prediction network not stable enough and could cause certain errors in the results;
(3) The data collected with remote sensing technology were greatly affected by factors such as sensors, shooting angles and atmosphere, which may cause the inversion errors.

## 5. Conclusions

This paper takes forest biomass inversion in Liaoning Province as the research object, extracts remote sensing factors through remote sensing data processing, combines topographic data (elevation, slope) and forest sample plot data, discusses the influencing factors of biomass in Liaoning Province and compares three different models to construct an optimal spatial distribution map of biomass in Liaoning Province. The main conclusions are as follows:

(1) The correlation between altitude and biomass was the highest, and the correlation coefficient was 0.404. The B2 band of Landsat 8 and the NDPI characteristic quantity of the vegetation index have important correlations with forest biomass inversion, and the correlation coefficients are −0.342 and 0.323, respectively. Among the characteristic factors extracted from Sentinel-1A radar images, seven factors were correlated with biomass (*p* < 0.05), and five factors were negatively correlated, except entropy.
(2) Comparing the inversion accuracy and training speed of the three models, the model based on the linear stepwise regression method has the fastest training speed, but the lowest model accuracy. The BP neural network has a strong fitting ability with complex data, a short training time and high model accuracy. Although the training time of the PSO improved neural network model is longer, the coefficient of determination between the predicted value and the measured value is the highest. The results show that there is a nonlinear relationship between the biomass and the strong correlation factors. The neural network model based on the particle swarm optimization algorithm is the best model for forest biomass inversion in the Liaoning region.
(3) According to the spatial distribution map of biomass, the areas with high forest biomass in Liaoning Province are mainly distributed in areas with high altitudes and steep slopes in the east and southwest, while the areas with low biomass are mainly concentrated in the plain areas with low altitudes and gentle slopes.

## Figures and Tables

**Table 1:** Correlation statistics between biomass and variables.

Factor	B2		B3	B4		B5		B6		B7	
Correlation coefficient	−0.342 **		−0.290 **	−0.302 **		−0.157 *		−0.337 **		−0.307 **	
Factor	ARVI		DVI	EVI		NDPI		NDVI		RVI	
Correlation coefficient	0.250 **		0.249 **	−0.228 **		0.323 **		0.313 **		0.322 **	
Factor	SVAI		VH	VV		altitude		slope		canopy closure	
Correlation coefficient	0.310 **		−0.070	−0.004		0.404 **		−0.015		0.328 **	
	Mean	Variance	Homogeneity	Contrast	Dissimilarity	Entropy	Second Moment	Correlation	ASM	MAX	Energy
B2	−0.310 **	−0.056	0.069	−0.036	−0.063	−0.087	0.083	0.107			
B3	−0.271 **	−0.092	0.162 *	−0.084	−0.137	−0.185 *	0.184 *	−0.040			
B4	−0.286 **	−0.052	0.171 *	−0.068	−0.163 *	−0.186 *	0.169 *	−0.007			
B5	−0.158 *	−0.046	0.004	0.001	−0.006	0.023	−0.063	0.004			
B6	−0.322 **	−0.168 *	0.145	−0.152	−0.159 *	−0.211 **	0.194 *	0.002			
B7	−0.285 **	−0.132	0.175 *	−0.156 *	−0.174 *	−0.201 *	0.184 *	0.037			
VH	−0.174 *	−0.191 *	−0.197 *	0.119	0.148	0.196 *		−0.053	−0.139	−0.136	−0.168 *
VV	−0.126	−0.148	−0.143	0.065	0.093	0.241 **		0.004	−0.136	−0.099	−0.176 *

Note: **, significantly correlated at the 0.01 level (two-sided); *, significantly correlated at the 0.05 level (two-sided).

**Table 2:** Stepwise regression model coefficients.

Model	Unstandardized Coefficient		Standardized Coefficient	t	Sig.
	B	Standard Error			
Constant	−243.422	126.015		−1.932	0.055
Altitude	0.090	0.025	0.276	3.533	0.001
Canopy closure	125.943	33.185	0.267	3.795	0.000
ARVI	−156.917	64.266	−0.472	−2.442	0.016
EVI	73.454	35.773	0.236	2.053	0.042
RVI	148.340	43.052	0.676	3.446	0.001
VVEntropy	34.447	18.391	0.127	1.873	0.063
B6Mean	24.306	13.932	0.417	1.745	0.083
B6	−245.049	93.419	−0.656	−2.623	0.010

**Table 3:** Comparison of prediction accuracy and training time of different models.

Model	R^2^	Time
Stepwise regression model	0.5468	0.0862 s
BP neural network model	0.78226	0.13 s
POS improved neural network model	0.83204	235.16 s

## Data Availability

Because I signed a confidentiality agreement with the National Center for Ecological Sciences, I cannot share the forest information. Sources of remote sensing data have been described above.
